# Deep Cervical-thoracic Osteolipoma Near the Brachial Plexus – Case Report and Literature Review

**DOI:** 10.1055/s-0042-1748967

**Published:** 2022-06-03

**Authors:** Leonardo Furtado Freitas, Márcio Luís Duarte, Fernando Silva Xavier, Fernanda Boldrini Assunção, Luis Roberto Mathias Júnior, Thiago Luiz Pereira Donoso Scoppetta

**Affiliations:** 1 Departmento de Neuroradiologia, Hospital São Camilo, São Paulo, SP, Brasil; 2 Departamento de Radiologia do Centro Radiológico e Especialidades Médicas São Gabriel, Praia Grande, SP, Brasil; 3 Departamento de Radiologia Musculoesquelética, Hospital São Camilo, São Paulo, SP, Brasil; 4 Departamento de Neurocirurgia, Hospital São Camilo, São Paulo, SP, Brasil

**Keywords:** biopsy, brachial plexus, magnetic resonance imaging, lipoma, tomography, x-ray computed

## Abstract

Osteolipoma is a rare benign variant of lipoma and constitutes less than 1% of all lipomas, presenting as a well-circumscribed painless mass. It is a tumor known to occur in several regions, usually intraosseous or adjacent to bone tissue, whose pathogenesis is still unclear. Imaging exams are useful in their evaluation and, mainly, in surgical planning, which consists of tumor excision. However, the definitive diagnosis of osteolipoma is made by histopathological examination. Although benign, osteolipomas can compress surrounding structures, leading to important symptomatology, as in this case reported in which it is in contact with the brachial plexus.

## Introduction

Plant first described osteolipoma in 1958. 1 Osteolipoma has several synonyms, including the following: ossifying lipoma, bone lipoma, lipoma with bone metaplasia, and osteomatous hamartoma. 2 This benign tumor, which usually presents as a well circumscribed painless mass, is characterized histologically by a lipoma with bone components—proliferation of mature adipose tissue with bony trabeculae. 1 3 It is a tumor commonly diagnosed in patients over 40 years of age. 1

Osteolipoma is a rare variant of lipoma and constitutes less than 1% of all lipomas. 4 5 It is a tumor known to occur in several regions, commonly in intra-bone structures (skull, spine, forearm, and knee) or adjacent to bone tissue (muscles and oral regions), with the head and neck region as its preferred site, 3 measuring between 0.8 and 0.9 cm. 4 However, we did not find studies with involvement near the brachial plexus.

When osteolipoma presents in intra-articular or just joint location, mechanical symptoms can lead to early symptomatology. 3 Due to the absence of specific radiological findings, the differential diagnosis for lesions with fatty and bone components is broad, such as teratoma, liposarcoma, and osteosarcoma. 3

We demonstrated a case of osteolipoma in the deep soft tissues of the left cervical-thoracic transition near the brachial plexus with imaging evaluation treated with marginal resection.

## Case Report

A 45-year-old man presented with pain and paresthesia on the left side of the neck for about 30 days, with intensification of the condition for a week and irradiation to the left upper limb, making it difficult to flex the fingers. On physical examination, he presented symptoms of left brachial plexopathy—root territory of C6–D1—and bicipital hyporeflexia.

**Fig. 1:**
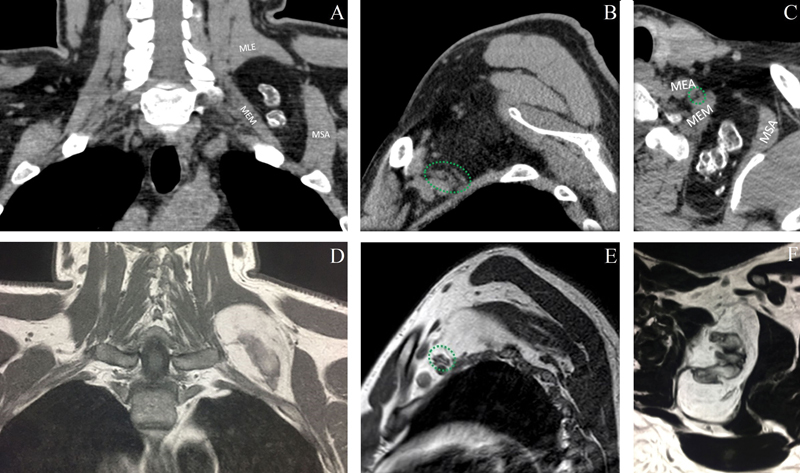
Computed tomography in coronal section in A, sagittal in B, and axial in C, demonstrating expansive formation located in the deep soft parts of the left paravertebral space, with attenuation similar to fat and coarse calcifications inside, in addition to thin septations in contact with the brachial plexus (green circle). Magnetic resonance in coronal cut T1 in D, sagittal T1 in E, and axial T2 in F, demonstrating encapsulated expansive formation located in the deep soft parts of the left paravertebral space with predominance of fat-like signal in all sequences with ossifications inside in contact with the brachial plexus (green circle). Abbreviations: MEA, anterior scalene muscle; MEM, medium scalena muscle; MLE, scapula lifter muscle; MSA, anterior serratus muscle.

**Fig. 2:**
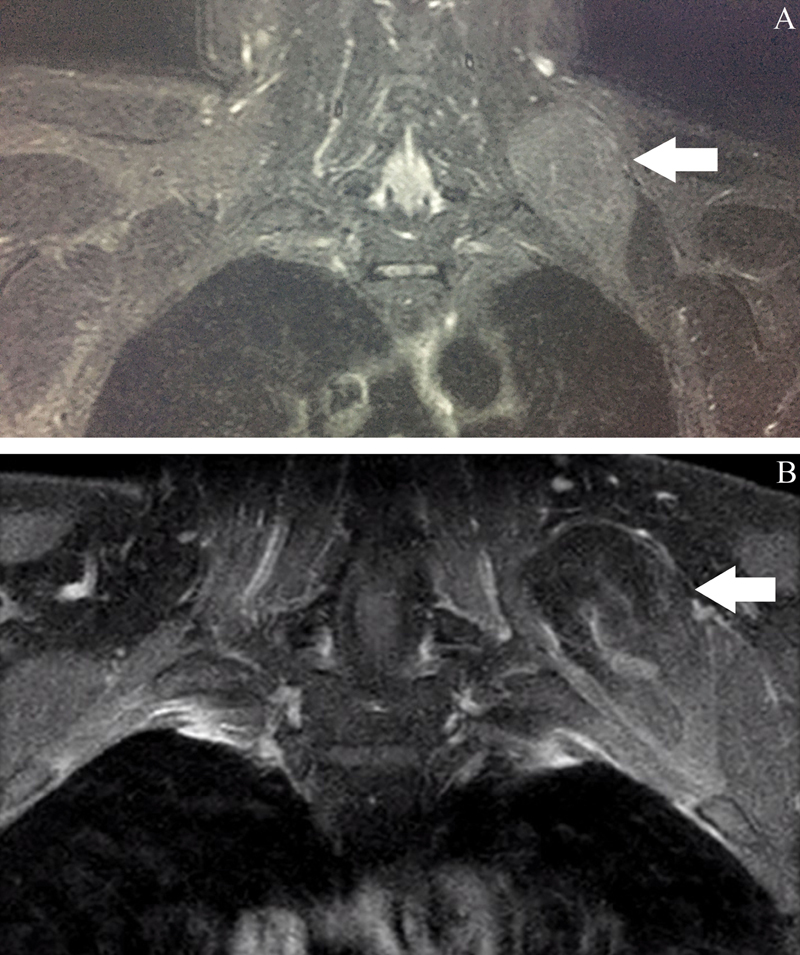
Magnetic resonance in coronal section in T2 stir sequence without contrast in A and T1 FAT SAT with contrast in B demonstrating that the expansive formation does not present significant contrast enhancement (white arrows).

**Fig. 3:**
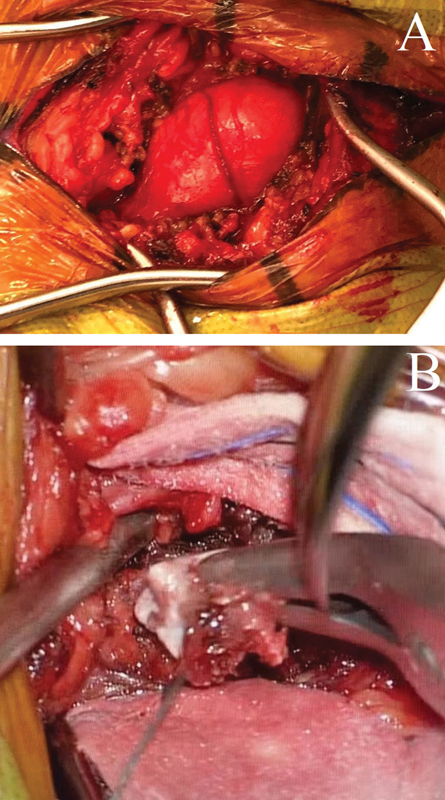
Intraoperatively demonstrating expansive formation in A and calcification in B. The lesion was completely restrained.

**Fig. 4:**
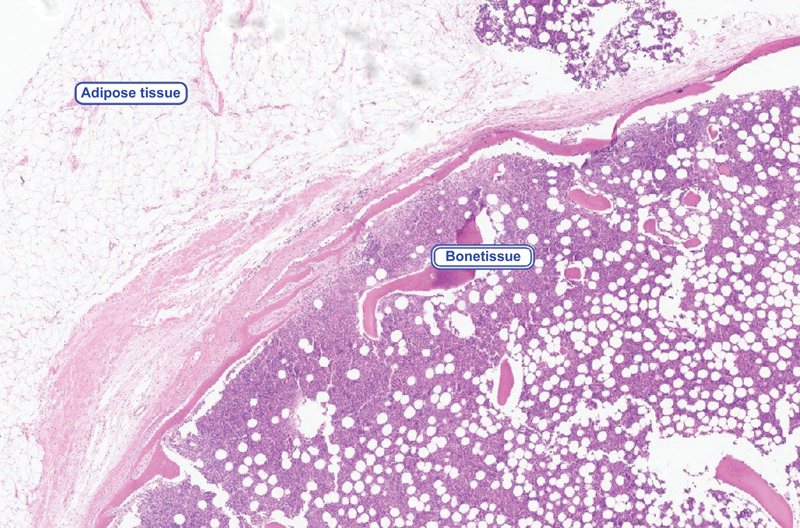
Photomicrograph of the anatomopathological study with hematoxylin & eosin staining and 20x magnification reveals a tumor consisting of mature adipose tissue with ossification areas with hematopoietic bone marrow elements without atypias, in addition to the absence of lipoblasts or signs of malignancy.

A computerized tomography (CT) was performed detecting relatively well-delimited expansive formation located in the deep soft parts of the cervical-thoracic transition on the left, with fat attenuation and surrounding ossifications, in addition to thin septations, near the brachial plexus ( Fig. 1 ). Magnetic resonance imaging (MRI) confirmed a lesion of a lipomatous nature, encapsulated and with ossifications inside, without contrast enhancement ( Figs. 1 and 2 ). It was performed with the anatomopathological evaluation by intraoperative freezing that evidenced mature mesenchymal neoplasia composed of benign adipocitary cells forming lobes in the delicate vascular web; presence of bone metaplasia with hematopoietic elements without atypia, without morphological signs of malignancy, confirming the diagnosis of osteolipoma, preceding exeresis of the lesion ( Figs. 3 and 4 ).

**Fig. 5:**
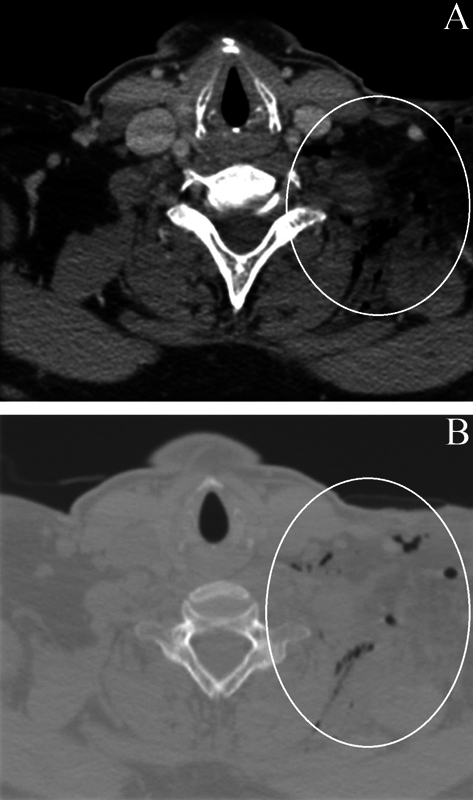
Postoperative computed tomography in axial section in bone window in A and soft-tissue window in B demonstrating densification of the deep planes of the surgical bed, as well as local unorganized liquid slides and sparse foci of gaseous component (white circles).

A CT performed 3 weeks after the surgery demonstrated densification of deep planes, unorganized liquid slides, and sparse foci of gas component, with complete macroscopic exeresis of the lesion ( Fig. 5 ). The patient did not present any motor or sensory deficit in the upper limbs in the postoperative follow-up in the following 8 months.

## Discussion

The pathogenesis of osteolipoma is still unclear, but some hypotheses have been proposed. 2 4 5 6 Castillo et al. assumed that osteolipoma is caused by the differentiation of multipotent mesenchyme cells within adipose tissue (metaplasia in preexisting lipoma). 1 This phenomenon may be caused by systemic and local metaplasia (trauma, chronic irritation, ischemia, and metabolic factors such as osteoinductor factors). 7 Blanshard and Veitch proposed that the transformation of osteoblasts into fibroblasts is driven by bone-inducing factors released by blood monocytes entering the adipose tissue. 7 Beranek proposed an alternative pathogenesis and suggested that, in some complex vascular tumors, such as a lipoma of vascular origin, the proliferation of two or three clear cell populations occurs simultaneously, originating from undifferentiated endothelial cells. 7 Fritchie et al. 4 reported cytogenetic analyses of three osteolipomas and concluded that translocations involving 12q13–15 in all cases were consistent with osteolipomas characteristics. Chromosomal abnormalities, such as translocations at 11q13, have also been reported. 1 5 Radiography does not commonly detect the lesion, 4 but it may reveal scattered calcifications and trabeculations. 2 6 Bone scintigraphy with fluorodeoxyglucose reveals a metabolically active lesion showing condroid calcifications associated with a large soft-tissue component showing fat density. 2 A CT is essential and provides an excellent visualization of calcified or ossified components and confirmation of the adjacent bone proximity, helping to determine the lesion's extent as well as surgical planning. 4 On CT, osteolipoma appears as a hypodense mass (adipose tissue) with hyperdense areas of bone tissue, but it may also reveal a mixed density. 1 6 8 On magnetic resonance imaging (MRI), osteolipoma is characterized by a high signal on T1-weighted and T2-weighted images referring to the lipomatous component with signal loss in sequences with fat saturation and areas ossified with medullary and bone cortical with low signal. 1 2 8 The postcontrast T1-weighted image shows coarse calcifications without significant nodular enhancement. 5 In addition to the precise topographic diagnosis and the evaluation of tumor extension, MRI allows the differentiation of osteolipoma from other tumors of the connective tissue. 1 In general, these radiological features described in the literature and demonstrated in this case are important to distinguish this tumor from more aggressive lesions containing fat, such as liposarcoma. 5

The definitive diagnosis of osteolipoma is made by histopathological examination, 6 as illustrated in the case presently described. The histological features of osteolipoma include predominantly mature adipose components with irregularly distributed mature lamellar bone tissue. 2 6 Differential diagnoses include congenital malformations of bones, teratomas, dermoids, calcified synovial cyst, tumor calcinosis, extra bone osteochondroma, ossifying myositis, ossifying fibromas, secondary ossification by trauma, liposarcoma with metaplastic alterations, nerve lipofibromatous hamartoma and osteosarcoma, which should all be considered in the differential diagnosis. 9 Early and precise treatment allows the decompression of the structures around the lesion and can avoid permanent sequelae, 4 8 10 as in the illustrated case. Surgical excision is usually chosen as a treatment for osteolipoma, 4 9 presenting a good prognosis. 5 6 9 Although relapses have not yet been reported, detailed monitoring and long-term follow-up are recommended due to the lack of clinical information. 5 7 We did not find studies describing osteolipoma with involvement near the brachial plexus in our searches, and, in this article, we report the first case diagnosed by imaging exams with histological confirmation.

There are few studies on this theme and, we emphasize that, despite the rarity of osteolipoma with involvement near the brachial plexus and its need for histological confirmation, this lesion should be in the differential diagnoses of lesions with calcifications/ossifications.
