# Supplementary material for: Deep Cervical-thoracic Osteolipoma Near the Brachial Plexus – Case Report and Literature Review
Source: Rev Bras Ortop (Sao Paulo). 2022 Jun 3;59(Suppl 1):e60–4. doi: 10.1055/s-0042-1748967 (PMC11254423; doi:10.1055/s-0042-1748967)
Supplement: Related texts

# Research article: Osteolipoma profundo cervicotorácico próximo ao plexo braquial – Relato de caso e revisão da literatura

**Keywords:** biópsia, imagem por ressonância magnética, lipoma, plexo braquial, tomografia computadorizada por raios X

## Abstract

O osteolipoma é uma rara variante benigna do lipoma e constitui menos de 1% de todos os lipomas, se apresentando como uma massa indolor bem circunscrita. É um tumor conhecido por ocorrer em várias regiões, comumente intraósseas ou adjacente ao tecido ósseo, cuja patogênese ainda não está clara. Os exames de imagem são úteis em sua avaliação e, principalmente, no planejamento cirúrgico, que consiste na exérese tumoral. Contudo, o diagnóstico definitivo do osteolipoma é realizado pelo exame histopatológico. Apesar de benigno, o osteolipoma pode comprimir estruturas ao seu redor, levando a importante sintomatologia como neste caso relatado em que está em contato com o plexo braquial.

## Introdução

Plant descreveu o osteolipoma pela primeira vez em 1958. 1 O osteolipoma tem vários sinônimos, incluindo os seguintes: lipoma ossificante, lipoma ósseo, lipoma com metaplasia óssea, e hamartoma osteolipomatoso. 2 Este tumor benigno, que normalmente se apresenta como uma massa indolor bem circunscrita, é caracterizado histologicamente por um lipoma com componentes ósseos – proliferação de tecido adiposo maduro com trabéculas ósseas. 1 3 É um tumor comumente diagnosticado em pacientes com mais de 40 anos de idade. 1

O osteolipoma é uma variante rara do lipoma e representa menos de 1% de todos os lipomas. 4 5 É um tumor conhecido por ocorrer em várias regiões, comumente em estruturas intraósseas (crânio, coluna vertebral, antebraço e joelho) ou adjacente ao tecido ósseo (músculos e regiões orais), cujo local preferencial é a região da cabeça e pescoço, 3 medindo entre 0.8 e 0.9 cm. 4 Contudo, não encontramos estudos com acometimento próximo ao plexo braquial.

Quando o osteolipoma se apresenta em localização intra-articular ou justa articular, os sintomas mecânicos podem levar à sintomatologia precoce. 3 Devido à ausência de achados radiológicos específicos, o diagnóstico diferencial para lesões com componentes gordurosos e ósseos é amplo, como por exemplo teratoma, lipossarcoma e osteossarcoma. 3

Demonstramos um caso de osteolipoma nas partes moles profundas da transição cervicotorácica à esquerda, próximas ao plexo braquial, com avaliação por exames de imagem tratado com ressecção marginal.

## Relato de Caso

Um paciente do sexo masculino, de 45 anos de idade, se queixou de dor e parestesia na face lateral esquerda do pescoço há cerca de 30 dias, com intensificação do quadro há uma semana e irradiação para o membro superior esquerdo, dificultando a flexão dos dedos. Ao exame físico, apresentava sintomas de plexopatia braquial à esquerda – território radicular de C6–D1–e hiporreflexia bicipital.

Foi realizada uma tomografia computadoriza (TC) que detectou formação expansiva relativamente bem delimitada e localizada nas partes moles profundas da transição cervicotorácica à esquerda, com atenuação de gordura e ossificações de permeio, além de finas septações, próxima ao plexo braquial ( Fig. 1 ). A ressonância magnética (RM) confirmou lesão de natureza lipomatosa, encapsulada e com ossificações no seu interior, sem realce por contraste ( Figs. 1 e 2 ). Foi realizada uma avaliação anatomopatológica por congelação intraoperatória que evidenciou neoplasia mesenquimal madura composta por células adipocitárias benignas formando lóbulos em meio à delicada trama vascular; presença de metaplasia óssea com elementos hematopoiéticos sem atipias nem indícios morfológicos de malignidade, confirmando o diagnóstico de osteolipoma, precedendo exérese da lesão ( Figs. 3 e 4 ).

**Fig. 1:** Tomografia computadorizada no corte coronal em A, sagital em B, e axial em C demonstrando formação expansiva localizada nas partes moles profundas do espaço paravertebral esquerdo, com atenuação semelhante a gordura, e calcificações grosseiras no seu interior, além de finas septações em contato com o plexo braquial (círculo verde). Ressonância magnética no corte coronal T1 em D, sagital T1 em E, e axial T2 em F demonstrando formação expansiva encapsulada localizada nas partes moles profundas do espaço paravertebral esquerdo com predomínio de sinal semelhante a gordura em todas as sequências com ossificações no seu interior em contato com o plexo braquial (círculo verde). Abreviaturas: MEA, músculo escaleno anterior; MEM, Músculo escaleno médio; MLE, músculo levantador da escápula; MSA, músculo serrátil anterior.

**Fig. 2:** Ressonância magnética no corte coronal na sequência T2 STIR sem contraste em A e T1 FAT SAT com contraste em B demonstrando que a formação expansiva não apresenta realce significativo pelo contraste (setas brancas).

**Fig. 3:** Intraoperatório demonstrando a formação expansiva em A e calcificação em B. A lesão foi ressecada em sua totalidade.

**Fig. 4:** Fotomicrografia do estudo anatomopatológico com coloração em hematoxilina & eosina e magnificação de 20x revela tumor constituído por tecido adiposo maduro com áreas de ossificação com elementos hematopoiéticos de medula óssea sem atipias, além da ausência de lipoblastos ou sinais de malignidade.

A TC realizada 3 semanas após a cirurgia demonstrou densificação dos planos profundos, lâminas líquidas não organizadas, e focos esparsos de componente gasoso, com exérese macroscópica completa da lesão ( Fig. 5 ). O paciente, não apresentou nenhum déficit motor ou sensorial nos membros superiores no acompanhamento pós-operatório nos 8 meses seguintes.

**Fig. 5:** Tomografia computadorizada pós-operatória no corte axial na janela óssea em A e janela para partes moles em B demonstrando densificação dos planos profundos do leito cirúrgico, assim como lâminas líquidas não organizadas locais e focos esparsos de componente gasoso (círculos brancos).

## Discussão

A patogênese do osteolipoma ainda não está clara, mas algumas hipóteses foram propostas. 2 4 5 6 Castillo et al. presumiram que um osteolipoma é causado pela diferenciação de células mesenquimais multipotentes dentro do tecido adiposo (metaplasia em lipoma pré-existente). 1 Esse fenômeno pode ser causado por metaplasia sistêmica e local (trauma, irritação crônica, isquemia e fatores metabólicos como fatores osteoindutores). 7 Blanshard e Veitch propuseram que a transformação de osteoblastos em fibroblastos é conduzida por fatores indutores de osso liberados por monócitos sanguíneos que entram no tecido adiposo. 7 Beranek propôs uma patogênese alternativa e sugeriu que, em alguns tumores vasculares complexos, como um lipoma de origem vascular, a proliferação de duas ou três populações de células claras ocorre simultaneamente, originadas de células endoteliais indiferenciadas. 7 Fritchie et al. 4 relataram análises citogenéticas de três osteolipomas e concluíram que as translocações envolvendo 12q13–15 em todos os casos eram consistentes com as características dos osteolipomas. Anormalidades cromossômicas, como translocações em 11q13, também foram relatadas. 1 5 A radiografia comumente não detecta a lesão, 4 mas pode revelar calcificações dispersas e trabeculações. 2 6 A cintilografia óssea com fluordesoxiglicose revela uma lesão metabolicamente ativa mostrando calcificações do tipo condroide associadas a um grande componente de tecido mole mostrando densidade de gordura. 2 A TC é essencial e proporciona uma excelente visualização dos componentes calcificados ou ossificados e confirmação da proximidade do osso adjacente, ajudando a determinar a extensão da lesão e o planejamento cirúrgico. 4 Na TC, o osteolipoma aparece como uma massa hipodensa (tecido adiposo) com áreas hiperdensas de tecido ósseo, mas também pode revelar uma densidade mista. 1 6 8 Na RM, o osteolipoma é caracterizado por alto sinal nas imagens ponderadas em T1 e T2 referente ao componente lipomatoso com queda de sinal nas sequências com saturação de gordura e áreas ossificadas com medular e cortical óssea de baixo sinal de permeio. 1 2 8 A imagem ponderada em T1 pós-contraste mostra calcificações grosseiras sem realce nodular significativo. 5 Além do diagnóstico topográfico preciso e da avaliação da extensão tumoral, a RM permite diferenciar o osteolipoma de outros tumores do tecido conjuntivo. 1 No geral, essas características radiológicas descritas na literatura e demonstradas neste caso são importantes para distinguir esse tumor de lesões mais agressivas contendo gordura, como o lipossarcoma. 5

O diagnóstico definitivo do osteolipoma é feito por exame histopatológico, 6 tal como ilustrado no caso discutido no presente artigo. As características histológicas do osteolipoma incluem componentes adiposos predominantemente maduros com tecido ósseo lamelar maduro irregularmente distribuído. 2 6 Os diagnósticos diferenciais incluem malformações congênitas dos ossos, teratomas, dermoides, cisto sinovial calcificado, calcinose tumoral, osteocondroma extra ósseo, miosite ossificante, fibromas ossificantes, ossificação secundária por trauma, lipossarcoma com alterações metaplásicas, hamartoma lipofibromatoso do nervo e osteossarcoma e devem ser considerados no diagnóstico diferencial. 9 O tratamento precoce e preciso permite a descompressão das estruturas ao redor da lesão, podendo evitar sequelas permanentes, 4 8 10 tais como no caso ilustrado. A excisão cirúrgica geralmente é escolhida como tratamento para o osteolipoma, 4 9 apresentando bom prognóstico. 5 6 9 Embora as recidivas ainda não tenham sido relatadas, o monitoramento detalhado e o acompanhamento em longo prazo são recomendados devido à falta de informações clínicas. 5 7 Não encontramos estudos descrevendo o osteolipoma com acometimento próximo ao plexo braquial em nossas buscas, e, neste artigo, relatamos o primeiro caso diagnosticado pelos exames de imagem com confirmação histológica.

Existem poucos estudos em relação a esse tema e, reforçamos que, apesar da raridade do osteolipoma com acometimento próximo ao plexo braquial e sua necessidade de confirmação histológica, esta lesão deve estar nos diagnósticos diferenciais de lesões com calcificações/ossificações.
